# Evaluation of chloroquine therapy for vivax and falciparum malaria in southern Sumatra, western Indonesia

**DOI:** 10.1186/1475-2875-9-52

**Published:** 2010-02-12

**Authors:** Inge Sutanto, Dedeh Endawati, Liem Hui Ling, Ferdinand Laihad, Rianto Setiabudy, J Kevin Baird

**Affiliations:** 1 Department of Parasitology Faculty of Medicine University of Indonesia, Jakarta, Indonesia; 2 National Agency for Drug and Food Control, Ministry of Health, Jakarta, Indonesia; 3 Post Graduate Study Programme, Faculty of Medicine, University of Indonesia, Jakarta, Indonesia; 4 Malaria Subdirectorate, Communicable Disease Control, Ministry of Health, Jakarta, Indonesia; 5 Department of Pharmacology Faculty of Medicine University of Indonesia, Jakarta, Indonesia; 6 Eijkman-Oxford Clinical Research Unit, Jakarta, Indonesia; 7 Centre for Tropical Medicine, Nuffield Department of Clinical Medicine, Oxford University, Oxford, UK

## Abstract

**Background:**

Chloroquine was used as first-line treatment for *Plasmodium falciparum *or *Plasmodium vivax *in Indonesia before the initial launch of artemisinin combination therapy in 2004. A study to evaluate efficacies of chloroquine against *P. falciparum *and *P. vivax *was undertaken at Lampung in southern Sumatra, western Indonesia in 2002.

**Methods:**

Patients infected by *P. falciparum *or *P. vivax *were treated with 25 mg/kg chloroquine base in three daily doses over 48 hr. Finger prick blood was collected on Days 0, 2, 3, 7, 14, 21 and 28 after starting drug administration. Whole blood chloroquine and its desethyl metabolite were measured on Days-0, -3 and -28, or on the day of recurrent parasitaemia.

**Results:**

42 patients infected by *P. falciparum *were enrolled, and 38 fullfilled criteria for per protocol analysis. Only six of 38 (16%) showed a response consistent with senstivity to chloroquine. 25 of 32 failures were confirmed resistant by demonstrating chloroquine levels on day of recurrence exceeding the minimally effective concentration (200 ng/mL whole blood). The 28-day cumulative incidence of resistance in *P. falciparum *was 68% (95% CI: 0.5260 - 0.8306). Thirty one patients infected by *P. vivax *were enrolled, and 23 were evaluable for per protocol analysis. 15 out of 23 (65%) subjects had persistent or recurrent parasitaemia. Measurement of chloroquine levels confirmed all treatment failures prior to Day-15 as resistant. Beyond Day-15, 4 of 7 recurrences also had drug levels above 100 ng/mL and were classified as resistant. The 28-day cumulative incidence of chloroquine resistance in *P. vivax *was 43% (95% CI: 0.2715 - 0.6384).

**Conclusion:**

These findings confirm persistantly high levels of resistance to chloroquine by *P. falciparum *in southern Sumatra, and suggest that high-grade and frequent resistance to chloroquine by *P. vivax *may be spreading westward in the Indonesia archipelago.

## Background

*Plasmodium falciparum *and *Plasmodium vivax *each cause an estimated one to two million cases of malaria in Indonesia each year (SEARO). In 2004, the Ministry Health of Indonesia abandoned chloroquine as first-line treatment for *P. falciparum *and adopted artemisinin-based combination therapy (ACT). Evidence of high levels of chloroquine resistance by *P. falciparum *from various areas of Indonesia drove this decision [1-7].

In contrast, high levels of chloroquine-resistant *P. vivax *were reported only in hyper- to holoendemic West Papua in the eastern part of Indonesia [6,8-10]. Even though evidence of this problem first appeared nearly 20 years ago, chloroquine remained the front-line therapy for vivax malaria. However, in 2007 the Ministry of Health adopted ACT for vivax malaria in some parts of West Papua in far eastern Indonesia. Evidence from the western islands of Indonesia between 1992 until 1998, revealed relatively low risk (approx. 10%) of chloroquine- resistant *P. vivax *[3,11-15]. Nonetheless, in 2008 national treatment policy was changed to apply artesunate-amodiaquine as first-line therapy for vivax malaria throughout Indonesia. However, that policy applies only to confirmed vivax malaria, and cases diagnosed clinically still receive chloroquine or sulphadoxine/pyrimethamine as first-line therapy. Moreover, widespread over-the-counter trade in chloroquine may persist even longer. Thus, although this study was undertaken prior to recent changes in malaria therapy policy, the findings nonetheless bear upon persisting contemporary treatment practices.

## Methods

### Study location

The study was conducted from June to September 2002 at Hanura Health Center in Lampung Province, southern Sumatra. This health center covers a population resident between 105° - 106° eastern longitude and 5° - 6° southern latitude at the southern-most tip of Sumatra island. Shrimp farming represented a major economic activity and the ponds used in this industry, when neglected and overgrown with algal mats, created breeding sites for *Anopheles sundaicus*, an efficient and important malaria vector in the region [16]. The rainy season in southern Sumatra occurs from September until April, whereas the rest of the year is relatively dry. Pribadi *et al *first reported chloroquine-resistant *P. falciparum *from this area in 1981 [2]. The health center was nonetheless using chloroquine for first-line treatment of both falciparum and vivax malaria at the time of this study.

### Study subjects

A protocol detailing the work reported here was reviewed and approved by the Ethics Committee of the Faculty of Medicine, University of Indonesia. Patients with fever or history of fever in the last 48 hours were screened for mono- infection by *P. falciparum *or *P. vivax *based on microscopic examination of Giemsa-stained thick blood films. Inclusion criteria for either infection was age ≥ 1 year, parasite count ≥ 1,000 asexual parasites/μL blood and patients willing to sign informed consent. The exclusion criteria were mixed infection of plasmodia, pregnancy (HCG urine test), complicated or severe malaria, or history of drug allergies. Qualified patients provided informed consent and were enrolled in the study.

The total dose of chloroquine was 25 mg base/kg body weight over 48 hr (Day-0 & Day-1: 10 mg/kb and Day-2: 5 mg/kg). All doses were directly observed by a member of the research team. If subjects vomited within the first 30 min after drug administration, the treatment was repeated. If subjects vomited a second time they were withdrawn from the study. A qualitative check on chloroquine consumption prior to visiting the health center was done by Dill-Glazko urine test [17], but test outcome was not a criterion for excluding patients from enrollment.

Subjects were asked to come back to the health center on Day-1, -2, -3, -7, -14, -21, and -28, or at any time they had fever. At each visit, except on Day-1, blood was collected for microscopic examination for parasites, as well as for measuring chloroquine level (100 μL dried on Whatman #1 filter paper). Axillary temperature was measured at each visit.

### Treatment response

Treatment outcomes for *P. falciparum *were classified as early treatment failure (ETF), late treatment failures (LTF) and adequate clinical and parasitological responses (ACPR) based on the WHO protocol [18]. In brief, the criteria for ETF is parasitaemia on Day-3 with axilary temperature ≥ 37.5°C or density of parasitaemia on Day-2 is higher than Day-0 (≥ 75%) or density of parasitaemia on Day-3 is ≥ 25% of Day-0 or clinical condition of subjects becomes severe on Day-1, Day-2 or Day-3 with positive parasitaemia. Patients were categorized as LTF if between Day-4 and Day-28 parasitaemia reappeared with an axillary temperature ≥ 37.5°C or when parasitaemia recurred on Day-7, -14, -21 and -28 with axillary temperature < 37.5°C or subjects suffers from severe condition with positive parasitaemia between Day-4 until Day-28. Patients were classified having ACPR if within 28 days follow up did not show any signs of ETF or LTF.

Gauging and classifying the treatment response by *P. vivax *was as applied elsewhere [19,20]. In brief, direct treatment failures occurred if parasitaemia ≥ 25% Day-0 persisted to Day-3. Early treatment failure occurred if parasitaemia at any level persisted until Day-4. A recurrence failure occurred if parasitaemia became subpatent but reappeared between Day-5 and Day-28 after initiating treatment, and appeared with >100 ng chloroquine and its major metabolite, desethyl-chloroquine (CQ+DCQ) per mL whole blood [21]. Infections were classified as sensitive to chloroquine if parasitaemia was not detectable by Day-3 and no recurrent parasitaemia occurred up to Day-28.

Subjects were withdrawn from the study if they failed to appear on scheduled follow-up visits, if they showed intercurrent parasitaemia by another species of *Plasmodium*, or if they had a recurrent parasitaemia by the same species but with chloroquine levels below the minimally effective concentration for that species (< 200 ng/mL or < 100 ng/mL for *P. falciparum *and *P. vivax*, respectively).

Alternative treament with quinine (3 × 10 mg/kg bw for 7 days) was offered to subjects having recurrent parasitaemia. *Plasmodium vivax *infected patients were also treated with primaquine (0.25 mg/kg per day × 14 d) at the end of follow up period or together with quinine when they failed. Outcome of or compliance to alternative therapies was not monitored.

### Chloroquine levels

Chloroquine measurements were done with the assistance of the U.S. Naval Medical Research Unit #2 in Jakarta, Indonesia using their high performance liquid chromatorgraphy (HPLC) instruments [22]. Whole blood total chloroquine levels consisted of the sum of CQ and DCQ, and was quantified on Day-0 (before treatment), Day-3, Day-28 or on the day of recurrent parasitaemia.

### Statistical analyses

Data were double-entered into an ExcellTM database, corrected, locked and entered into an SPSS version 16.0 for uni- and bi-variate analysis applying p ≤ 0.05 as the limit of statistical significance. The cumulative risk of treatment failure was estimated using the life table method as described elsewhere [8], and 95% confidence intervals were calculated with Stata software

## Results

### Screening patients

3,154 patients came to the health center between June and September 2002, and 676 (21%) were screened for malaria by microscopic examination of Giemsa-stained blood films, of which 392 (58%) were found to be parasitaemic. These were *P. falciparum *(53% = 207/392), *P. vivax *(43% = 168/392) and mixed infection of *P. falciparum *and *P. vivax *(4% = 17/392). Confirmed malaria thus accounted for approximately 10% of patients seeking medical attention at this health center.

Only 82 (39%) of 207 *P. falciparum*-infected patients fullfilled the inclusion criteria. 123 had parasite counts of less than 1,000/μL blood, one had a positive pregnancy test and another declined informed consent. 42 of the eligible subject were randomized to this study and received chloroquine therapy. The remaining 40 were randomized to another protocol with sulphadoxine-pyrimethamine, and will be reported separately. Among the patients with *P. vivax*, only 31 of 137 (23%) had >1,000 asexual forms/μL, and all of these were enrolled in the current study.

### Demographic, clinical and parasitological characteristics

The sex ratio in both groups (*P. falciparum *and *P. vivax*) was comparable (Table 1). The age of *P. vivax*-infected subjects was not distinct from *P. falciparum*-infected subjects. On the day of enrolment, 62% and 48% of *P. falciparum *and *P. vivax *subjects were febrile, respectively. The geometric mean of parasite density was higher in *P. falciparum *(8,759/μL) than *P. vivax *(5,468/μL) subjects (Table 1). In both groups about one quarter of subjects had positive Dill-Glazko urine tests for chloroquine prior to supervised treatment.

**Table 1:** Demographic & clinical characteristics of subjects

Characteristic	*P. falciparum*	*P. vivax*
Number of subjects	42	31

Age (years)(median) (inter quartile range)	12 (7 - 17)	12.8 (1 - 60)

Male (percentage)	18 (43%)	14 (45%)

Body temperature on Day-0 (°C)(mean)(range)	37.8 (35.2 - 39.8)	37.6 (35.3 - 39.9)

Feverish patients on Day-0 (%)	26 (62%)	15 (48%)

Positive Dill Glazko in urine on Day-0 (%)	10 (24%)	8 (26%)

Geometric mean of parasite density on Day-0 (range)	8,758/μL (1,000-62,720)	5,468/μL (1,240 -41,720)

### Whole blood chloroquine levels

33 of 42 enrolled subjects infected by *P. falciparum *had chloroquine levels measured. On the day of enrollment, 15 of the 33 subjects (46%) had chloroquine in blood between 67 to 267 ng/mL (3 of these were above the minimally effective concentration of 200 ng/mL). There was no correlation between chloroquine level on Day-0 and Day-3 (Pearson correlation: r = 0.014, p value = 0.937). Furthermore, neither CQ+DCQ level on Day-0 nor density of parasitaemia on Day-0 correlated with treatment failure (Fisher exact test: CQ Day-0: p value = 0.686, OR = 1.417, 95% CI = 0.259 - 7.763; Parasite density Day-0: p value = 1.000, OR = 1.000, 95% CI = 0.175 -5.710).

Chloroquine blood levels were measured before supervised treatment in 23 of the 31 *P. vivax*-infected subjects, and 14 (65%) had chloroquine levels between 92 ng/mL to 392 ng/mL, all of these had CQ + DCQ above minimally inhibitory concentration of ≥ 100 ng/mL except one (92 ng/mL). There was no correlation between CQ+DCQ level on Day-0 and Day-3 (Pearson correlation: r = 0.007, p value = 0.974). Neither CQ+DCQ level on Day-0 (Fisher exact: p value = 0.400, OR = 2.625, 95% CI = 0.395 - 17.458) nor density of parasitaemia on Day-0 (Fisher exact: p value = 0.667, OR = 1.905, 95% CI = 0.330 - 11.009) were associated with treatment failure.

**Figure 1:**
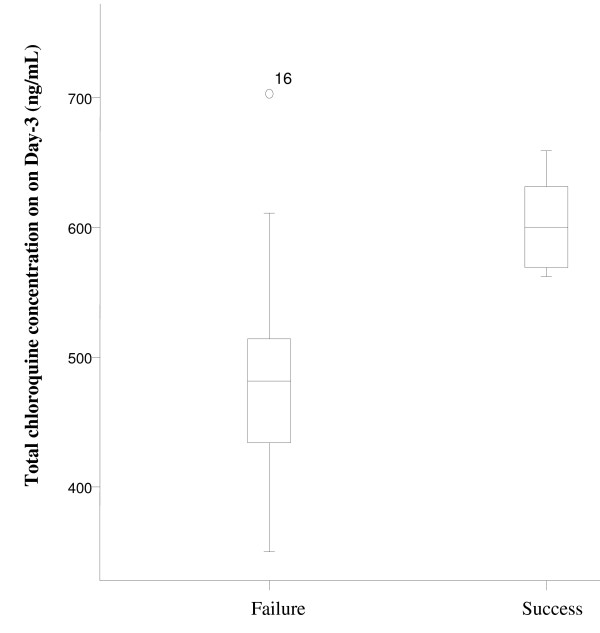
**Chloroquine level on Day-3 of *P. falciparum *subjects**. The median chloroquine level on Day-3 was significantly lower in failed than succeed groups of *P. falciparum *subjects (Mann-Whitney test, p = 0.001). Case number 16 was the outlier of failed subject with chloroquine level of 703 ng/mL.

**Figure 2:**
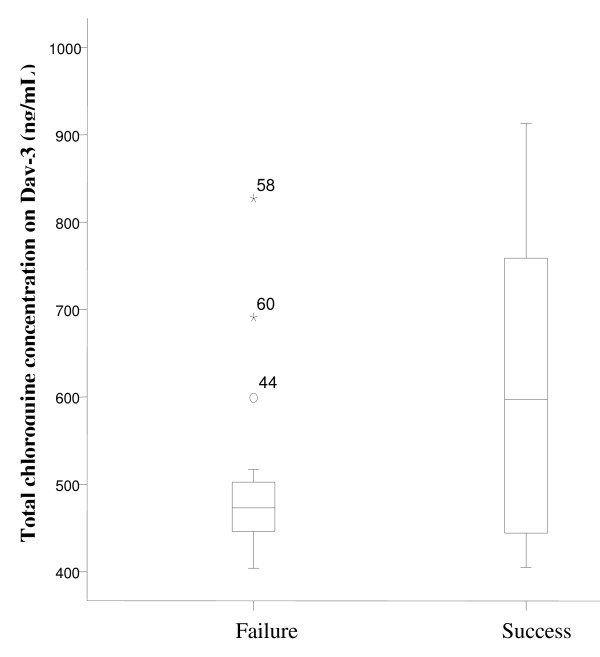
**Chloroquine level on Day-3 of *P. vivax *subjects**. The median chloroquine level on Day-3 was indifferent in failed and succeed groups of *P. vivax *subjects (Mann-Whitney test, p = 0.254). Case number 44, 60 & 58 were the outliers of failed subject with chloroquine level of 599, 691 and 827 ng/mL, respectively.

The risk of treatment failure with relatively low peak levels of chloroquine (Day-3) was assessed by comparing the median of total chloroquine level among failed and successfully treated subjects. In 33 *P. falciparum *subjects, chloroquine levels on Day-3 were significantly higher in successfull than failed subjects (Mann-Whitney: sums rank of success versus failures were 379 vs 182, p value = 0.001) (Figure 1). Nevertheless, in 23 *P. vivax *subjects the differences were not significant (Mann-Whitney: sums rank of success versus failures were 162 vs 114, p value = 0.245) (see Figure 2). This smaller sample may lack adequate power to detect such differences.

### 28 day test outcomes

Among 42 subjects infected by *P. falciparum*, 38 were evaluated based on per protocol analysis. Two were withdrawn due to mixed infections (Day 14 & 21), and two subjects did not reappear at the health center after Day-3 and -21. 16% (6/38) of failures occurred during peak drug levels (Day-2: 5 subjects; Day-3: 1 subject). Another nine (24%) failures occurred on Day-7, so that a total of 40% of treatment failures with *P. falciparum *occured within the first week. During Week-2 another 13 subjects (34%) had recurrent parasitaemia, and during Week-3 and Week-4 this occurred among three (8%) and one (3%) subject, respectively. The total proportion of chloroquine treatment failures was 84% (32/38) among *P. falciparum*-infected subjects.

Among 31 *P. vivax *subjects, 23 were evaluated based on per protocol analysis. Three dropped out (2 on Day-7 and 1 on Day-21) without citing reasons, and five withdrew due to intercurrent infection of *P. falciparum *(three on Day-21 and two on Day-28). Among subjects experiencing treament failure, one showed increasing numbers of parasites on Day-2 (6,640 vs. 7,120 parasites/μL) and was classified as a direct treatment failure. Three subjects showed decreased parasitaemia from Day-0 to Day-2 or Day-3 (2,680 vs. 280 parasites/μL; 2,200 vs. 360 parasites/μL; & 2,640 vs. 200 parasites/μL). These three parasitaemias persisted to Day-7 and were classified as early treament failures. Eleven subjects had recurrent parasitaemia by Day-14 (4), Day-21 (6) and Day-28 (1). These were classified as recurrence treatment failures. The crude proportion of chloroquine treatment failures among *P. vivax*-infected subjects was 65% (15/23).

### Life table analyses

Life table analysis does not censor withdrawn subjects until the day of loss. In *P. falciparum*-infected subjects, 10% (6 subjects) failed at peak drug levels on Day-2 &-3, but only four of them (three on Day-2 and one on Day-3) had chloroquine levels that confirmed resistance. The other 2 subjects on Day-2 did not have available blood blots for drug measurement (Table 2). All failures on Day-7 (9 participants) were resistant, and the 7-day cumulative incidence of failure was 33%. Furthermore, on Day-14, 21 & 28 some subjects could not be confirmed as resistant (three, one and one subject, respectively) due to CQ+DCQ level being < 200 ng/mL. At Day-14 the estimated cumulative incidence of therapeutic failure was 61%. This estimate was 68% by Day-28 (95% CI: 0.5260 - 0.8306). Among *P. vivax-*infected subjects, all treatment failures up to Day-14 were confirmed resistant (8 subjects) (Table 3), and beyond Day-14 four of seven recurrent parasitaemias were confirmed as resistant by CQ+DCQ levels >100 ng/mL. The cumulative incidence of confirmed resistance was estimated to be 3% at Day-3, 13% at Day-7, 27% at Day-14, and 43% at Day-28 (95% CI: 0.2715 - 0.6384).

**Table 2:** Life table estimation of cumulative incidence of chloroquine resistance in *P. falciparum*

Day	N	I	W	IR	CIR	95% Confidence Interval
0	42	0	0	0	0	------

2	42	3	2	0.0731	0.0731	0.0242 - 0.2100

3	37	1	1	0.0274	0.0984	0.0382 - 0.2418

7	35	9	0	0.2571	0.3302	0.2069 - 0.5004

14	26	10	4	0.4167	0.6093	0.4578 - 0.7640

21	12	2	3	0.1905	0.6837	0.5260 - 0.8306

28	7	0	1	0	0.6837	0.5260 - 0.8306

D = Day of the test

N = number of subjects remaining at risk

I = incident of resistant cases

W = withdrawals due to intercurrent parasitaemia by *P. vivax *or loss to follow up or

parasitaemia positive with CQ level <200 ng/mL

IR = Interval risk (i{N-(w/2)}^-1^

CIR_n _= cumulative incidence of resistant case

= 1-(1-IR_n _× 1-CIR_n-1_) where n is day of test and n-1 is the prior interval, w.g. for calculating CIR day 14, use IR day 14 and CIR day 7

**Table 3:** Life table estimation of cumulative incidence of chloroquine resistance in *P. vivax*

Day	N	I	W	IR	CIR	95% Confidence Interval
0	31	0	0	0	0	----

2	31	1	0	0.0333	0.0323	0.0046 - 0.2077

3	30	0	0	0	0.0323	0.0046 - 0.2077

7	30	3	2	0.1034	0.1324	0.0518 - 0.3153

14	25	4	0	0.16	0.2712	0.1457 - 0.4703

21	21	4	6	0.2222	0.4331	0.2715 - 0.6384

28	11	0	3	0	0.4331	0.2715 - 0.6384

D = Day of the test

N = number of subjects remaining at risk

I = incident of resistant cases

W = withdrawals due to intercurrent parasitaemia by *P. falciparum *or loss to follow up or

parasitaemia positive with CQ level < 100 ng/mL

IR = Interval risk (i{N-(w/2)}^-1^

CIR_n _= cumulative incidence of resistant case

= 1-(1-IR_n _× 1-CIR_n-1_) where n is day of test and n-1 is the prior interval, w.g. for calculating CIR day 14, use IR day 14 and CIR day 7

## Discussion

This small study documented the 28-day cumulative incidence of chloroquine-resistance in *P. falciparum *(68%) and *P. vivax *(43%) infections acquired in southern Sumatra in western Indonesia in 2002. In earlier studies in this region, chloroquine-resistant *P. falciparum *[2] and *Plasmodium malariae *[23] were reported, although the latter diagnosis has been debated [24]. Nonetheless, at the time of our work, chloroquine remained first-line therapy for falciparum malaria throughout Indonesia. This was finally changed in 2004 to artesunate combined with amodiaquine, although even today only for confirmed infections. The very high levels of resistance documented here affirm the necessity of that change in policy and practice, but these data also serve as a basis for comparison with the same phenomenon in vivax malaria, against which chloroquine remains in wide use.

Studies conducted in Indonesia between 1992 and 2001 documented chloroquine-resistant *P. vivax *(CRPV) occurring at > 40% risk in Indonesian New Guinea (Papua, formerly Irian Jaya) [6,8-10]. Similar surveys in central and western Indonesia, however, revealed relatively low risk (<20%) [3,11-15]. The apparent isolation of high risk of CRPV to Indonesian New Guinea encouraged health authorities to maintain chloroquine as the national first-line therapy for vivax malaria. This was even true in Papua until 2007 when a location-specific treatment policy trial was instituted in the area of Timika, where more recent studies also documented very high risk of CRPV [10,25]. That first line therapy for Timika is dihydroartemisinin combined with piperaquine, and has been under evaluation by the authorities.

Very recently, in 2008, the Ministry of Health reached a decision to recommend artesunate-amodiaquine as first-line therapy against vivax malaria as national treatment policy. This combination therapy is now recommended as first-line treatment for either *P. falciparum *or *P. vivax *across Indonesia. However, many diagnoses of malaria in Indonesia today are made clinically or non-specifically, and such cases still receive chloroquine or sulphadoxine/pyrimethamine therapies. No study has yet gauged the extent and rate of specific diagnoses and ACT treatment, and how these practices impact metrics of malaria risk in any given area.

The findings in the current study, and another recently reported trial [19], corroborate the description of the CRPV situation on the Indonesian archipelago as worsening. In our other study on Alor Island in the eastern Lesser Sundas Archipelago, the 28-day risk of CRPV was estimated at 56%. The current study, approximately 3,000 km west of Papua, found a 43% risk of CRPV, well above the prior studies in western Indonesia ranging from 0% to 20% [3,11-15]. Recent work from Myanmar [26] also suggests a westward encroachment of CRPV. With India representing the nexus of the global vivax malaria burden [27], this trend merits aggressive surveillance.

Surveillance of CRPV has been hampered by the low priority assigned to vivax malaria by donors, sponsors and health agencies. More than two decades after the emergence of evidence of a serious resistance problem, the number of surveys of CRPV in that interim anywhere in the world numbers less than a few dozen. Another inhibiting factor has been the unavailability of a standardized testing procedure, or the application of procedures rooted in those for falciparum malaria and poorly suited for vivax malaria. The analytical methodology applied here and in another recent study [19] derived from earlier work [21], and the terminology classifying outcomes as sensitive or direct, early or recurrent treatment failures is our own. These methods and terminologies are useful and analytically sound in the narrow context of characterizing resistance to chloroquine by asexual blood stages of *P. vivax*. The rationale underpinning these approaches hinge upon pharmacokinetics unique to chloroquine and upon the specific sensitivities of *P. vivax *isolates that may be considered unambiguously a sensitive phenotype.

In this study, 77% of otherwise eligible subjects with vivax malaria were excluded on the basis of an arbitrary minimal parasite count of 1,000/μL. This experience encouraged us to subsequently abandon such a potentially powerful source of confounding by sample bias. We acknowledge that such systemic sample bias may in part explain the apparently high rate of resistance recorded in this study. A sample that included the median parasitemia in this community would have very likely produced a lower cumulative incidence of recurrences and more truly represented risk of therapeutic failure in the study area.

## Conclusions

The findings in this study represent only a few dozen patients from the catchment area of a single health center in southern Sumatra, Indonesia. Further surveillance for CRPV should be carried out in the region to more reliably characterize risk of CRPV. This is true even though chloroquine is no longer first-line therapy for vivax malaria in Indonesia. Some years may pass before existing stocks of chloroquine become exhausted and the drug is likely to see fairly extensive use, including through private marketers catering to a brisk over-the-counter trade in this drug.

## Competing interests

The authors declare that they have no competing interests.

## Authors' contributions

IS participated in study design, data collection, analysed data and drafted manuscript; FL participated in study design and data collection; DE carried out CQ and DCQ measurement with HPLC and analysed the data, LHI participated in sample and data collection in the field; RS participated in data analysis and interpretation, JKB participated in data interpretation, manuscript writing, and critical review. All authors read critically and agree the manuscript to be submitted.
